# Neurogenesis reunited

**DOI:** 10.7554/eLife.14955

**Published:** 2016-03-15

**Authors:** Matthias Landgraf

**Affiliations:** Department of Zoology, University of Cambridge, Cambridge, United Kingdomml10006@cam.ac.uk

**Keywords:** neuroblast, neuronal lineages, neuronal development, HOX genes, transcription factors, <i>D. melanogaster</i>

## Abstract

Experiments on fruit flies are shedding new light on the evolution and development of the nervous system in metamorphosing insects.

**Related research article** Lacin H, Truman JW. 2016. Lineage mapping identifies molecular and architectural similarities between the larval and adult *Drosophila* central nervous system. *eLife* **5**:e13399. doi: 10.7554/eLife.13399

**Image** Lineages of nerve cells (green and pink) in nervous system of an early-stage fruit fly larva

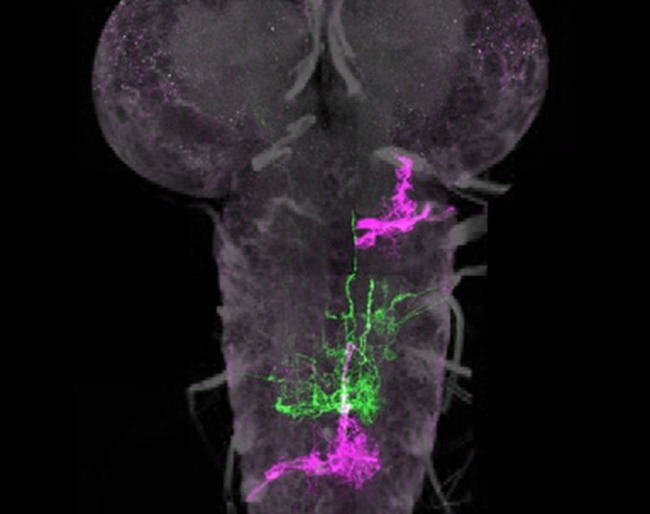

Metamorphosis is a wonderful and curious process in which animals undergo a transformation from one form and lifestyle to another. It is thought that insects that undergo metamorphosis – which include flies, beetles, bees and butterflies – make up the majority of animal species on Earth. However, the first insects to evolve did not undergo this process. Instead, much like modern-day silverfish, these earliest insects had embryos that developed into essentially miniature versions of the adult forms. Metamorphosing insects evolved later and have embryos that develop into simple larvae. These larvae move, feed and grow until they reach a critical size, at which point they form a pupa and undergo metamorphosis. The transformation involves many of the tissues that had developed in the embryo being broken down so that new adult structures – such as legs, wings, eyes, antennae and genitalia – can form.

The metamorphosis of an insect’s body is paralleled by changes to its nervous system. The nerve cells required for the simple larva are born in the embryo from other cells called neuroblasts. This process, which is called neurogenesis, also occurs in a late stage larva when new nerve cells are needed to build the adult’s nervous system.

In the early 1990s, it was elegantly demonstrated that the active neuroblasts in larvae of the fruit fly *Drosophila melanogaster* are in fact embryonic neuroblasts that have been re-activated (Prokop and Technau, 1991). However many researchers study neurogenesis in either the embryo or the larva, but not both. This means that for decades these two fields of research have remained largely disconnected, as if they concerned two different beasts. However, last year, Gerhard Technau and colleagues at the University of Mainz started to bridge this gap by studying these two phases of neurogenesis in *D. melanogaster* (Birkholz et al., 2015). Now, in eLife, Haluk Lacin and James Truman of the Janelia Research Campus report how they have built on this work to complete the job (Lacin and Truman, 2016).

The nervous systems of different insect species develop from ‘ground plans’ that are found in each segment of an insect’s body and defined by neuroblasts. Figuring out how these different ground plans are modified is essential if we are to understand how the nervous systems of insects evolved and develop. Metamorphosing insects, such as *D. melanogaster*, present an interesting riddle. The fruit fly larva is a small, worm-like creature and most of its body segments perform very similar roles. About 32 neuroblasts in the embryo produce the nerve cells needed for the comparatively simple nervous system in the thorax and abdomen of a larva (Bate, 1976; Birkholz et al., 2013). When neurogenesis resumes in a late stage larvae, 23 neuroblasts whir into action in the thorax, but only a few are re-activated in the abdomen (Truman and Bate, 1988). This larval neurogenesis produces about 90% of the nerve cells found in an adult fly, including those that control its legs and wings.

The work at Mainz and Janelia has now revealed which neuroblasts are exclusively active in the embryo, and which become re-activated in thorax and abdomen of the larva. Technau and colleagues used an approach called the “Flybow” technique to track neuroblast cells (plus the cells descended from these neuroblasts) from the embryo to the late-stage larva just before metamorphosis (Birkholz et al., 2015; Hadjieconomou et al., 2011). Lacin and Truman, on the other hand, used new genetic tools that allowed them to follow lineages of specific neuroblasts and their descendants all the way into the pupal and adult stages (Lacin and Truman, 2016; Awasaki et al., 2014).

While both studies largely agreed, there are some discrepancies. For example, Lacin and Truman discovered a new neuroblast in the larva’s thorax. It appears that this neuroblast (which they named NB5-7) most likely arose via a duplication of a neighbouring neuroblast (called NB5-4). What is more, while both of these neuroblasts produce similar nerve cells associated with motor control of the legs in adult flies (Harris et al., 2015), only NB5-4 also gives rise to cells in the embryo. Together these data point to NB5-7 being a recent evolutionary modification of the basic neuroblast ground plan, and producing the additional cells for the adult nervous system that might boost the control of leg movements. Lacin and Truman also demonstrate that the presence of the NB5-4 neuroblasts depended on specific genes that control the development of animal body plans – the so-called HOX*﻿* genes (see also Bello et al., 2003).

So how should we now look at neurogenesis in metamorphosing insects? It is possible that the evolution of metamorphosis ushered in a separate mode of nervous system development that is specific to the adult stage. Alternatively, the pause in nerve cell birth that is seen at the end of embryonic development may simply be just that: a pause. Drawing on a wealth of data, Lacin and Truman show that, for *D. melanogaster*, the pause is just a pause. Moreover, it has now become clear that – rather than being two distinct phases – larval neurogenesis appears to simply resume where embryonic production left off. For example, some neuroblasts in the late embryo produce nerve cells ready for the adult’s nervous system, which remain mostly undifferentiated until the larval stages. When the same neuroblasts become active again in the larva, they then produce new nerve cells that are similar to those last made in the embryo. Finally, although some details remain to be ironed out, by casting embryonic and larval neurogenesis in *Drosophila* as a continuum, Lacin and Truman have opened new avenues for studying the development and evolution of nervous systems.

## References

[bib1] Awasaki T, Kao C-F, Lee Y-J, Yang C-P, Huang Y, Pfeiffer BD, Luan H, Jing X, Huang Y-F, He Y, Schroeder MD, Kuzin A, Brody T, Zugates CT, Odenwald WF, Lee T (2014). Making *Drosophila* lineage–restricted drivers via patterned recombination in neuroblasts. Nature Neuroscience.

[bib2] Bate CM (1976). Embryogenesis of an insect nervous system. I. A map of the thoracic and abdominal neuroblasts in *Locusta migratoria*. Journal of Embryology and Experimental Morphology.

[bib3] Bello BC, Hirth F, Gould AP (2003). A pulse of the *Drosophila* Hox protein Abdominal-A schedules the end of neural proliferation via neuroblast apoptosis. Neuron.

[bib4] Birkholz O, Rickert C, Berger C, Urbach R, Technau GM (2013). Neuroblast pattern and identity in the *Drosophila* tail region and role of *doublesex* in the survival of sex-specific precursors. Development.

[bib5] Birkholz O, Rickert C, Nowak J, Coban IC, Technau GM (2015). Bridging the gap between postembryonic cell lineages and identified embryonic neuroblasts in the ventral nerve cord of *Drosophila melanogaster*. Biology Open.

[bib6] Hadjieconomou D, Rotkopf S, Alexandre C, Bell DM, Dickson BJ, Salecker I (2011). Flybow: genetic multicolor cell labeling for neural circuit analysis in *Drosophila melanogaster*. Nature Methods.

[bib7] Harris RM, Pfeiffer BD, Rubin GM, Truman JW (2015). Neuron hemilineages provide the functional ground plan for the *Drosophila* ventral nervous system. eLife.

[bib8] Lacin H, Truman JW (2016). Lineage mapping identifies molecular and architectural similarities between the larval and adult *Drosophila *central nervous system. eLife.

[bib9] Prokop A, Technau GM (1991). The origin of postembryonic neuroblasts in the ventral nerve cord of *Drosophila melanogaster*. Development.

[bib10] Truman JW, Bate M (1988). Spatial and temporal patterns of neurogenesis in the central nervous system of *Drosophila melanogaster*. Developmental Biology.

